# Organizational reputation: does it mediate the effect of *employer brand attractiveness* on *intention to apply* in Indonesia?

**DOI:** 10.1016/j.heliyon.2022.e09208

**Published:** 2022-03-29

**Authors:** Pantius D. Soeling, Sesilia Dhea Ajeng Arsanti, Fibria Indriati

**Affiliations:** a Department of Business Administration, Faculty of Administrative Sciences, Universitas Indonesia, Indonesia; b Astra Credit Company, Indonesia

**Keywords:** Employer brand attractiveness, Organizational reputation, Intention to apply, Generation millennials, Employer branding

## Abstract

This study aims to examine the role of organizational reputation in mediating the influence of employer brand attractiveness on intention to apply. Organizational reputation has an essential role in attracting potential talent to apply for an organization, as organizational reputation is an intangible and valuable resource to gain the competitive advantage that shows the working atmosphere in the organization. The study investigated organizational reputation as a mediating variable on the relationship between employer brand attractiveness as an independent variable and intention to apply as a dependent variable. Data were collected using a self-administered questionnaire that was distributed to 425 respondents. Respondents for this study were final-grade students from public universities in Indonesia. The path analysis technique was used to analyse the data. The result shows that employer attractiveness significantly influences the intention to apply. The result also reveals that employer brand attractiveness significantly affects the organizational reputation. Meanwhile, organizational reputation does not influence the intention to apply. Therefore, organizational reputation does not mediate the influence of employer brand attractiveness on the intention to apply. From this study, organizations can learn how to design programs that can improve employer brand attractiveness, particularly among gen millennials.

## 1 Introduction

Human resource (HR) is an essential asset of the organization in carrying out operational activities and achieving strategic goals, vision and mission. HR is a critical aspect for competitive advantage and a company's leading investment (Sivertzen et al., 2013). Therefore, if a company can find and retain better and more qualified talent than competitors, it can achieve profits (Boxall, 1996). McKinsey introduced the talent war in 1998, where he stated that better talent was worth fighting for (Chambers et al., 1998). Talents are a source of life for any organization or company, and every company realizes that talent and talent skills are the most critical drivers for company success (Maurya and Agarwal, 2018). The employee recruitment process is an initial effort to attract and obtain qualified employees to achieve a company's vision, mission and goals. According to Mullins (2005), the recruitment process helps select and place the right people in the right jobs, indicating high-performing organizations. In recruiting potential resources, a company needs to carry out effective and efficient strategies to increase the quantity and quality of job applicants, by creating interest in a company (Barber, 1998 in Kavitha and Srinivasan, 2012). The initial stage in recruiting workers is to attract applicants into a company (Carless, 2007). Attracting applicants is a crucial stage because without it, the following process of recruiting potential resources will not be achieved, including selection (Acarlar and Bilgic, 2012). According to Gomes and Neves (2011), a company must understand the factors related to the intention to apply for a job because recruitment needs to be carried out effectively. The initial stage in recruiting workers is to attract applicants into a company (Carless, 2007). Cappelli (2001) stated that in the business world with fierce and open competition, employer branding and organizational reputation are crucial in attracting the best employees. Employer branding and organizational reputation are often used to describe what job seekers emphasize when applying for jobs (Sivertzen et al., 2013). Additionally, employer branding is used to increase employer attractiveness and enhance company reputation (Sivertzen et al., 2013). Job seekers often consider several organizations when they apply for the job, and they can use organizational reputation as a source of information about working conditions in various organizations (Cable and Turban, 2003). According to Walsh and Beatty (2007), organizational reputation is considered one of the intangible and valuable resources that can contribute to achieving competitive advantage and is one of the important things job seekers consider when applying for a job. Melo and Garrido (2012) stated that an excellent organizational reputation could be a magnet for attracting and retaining potential employees. One effort that can be performed to increase interest is to build a positive organizational reputation through employer brand attractiveness. To recruit workers, a company needs to understand the factors that increase employer attractiveness, in order to increase job applicants. Employer attractiveness is a benefit seen by potential employees in jobs in specific organizations (Berthon, 2005). Employer attractiveness relates to how a company tries strategically to exploit the organization's strength to attract applicants. The workforce is currently dominated by Generation Y, also known as millennials. Millennials are people born between 1981 – 2000 (VanMeter et al., 2015). Generation Y is more interested in working in transparent organizations where the vision, mission, values, operations and conflicts within a company are shared openly. Suppose a company wants to attract and retain Generation Y employees. In that case, the organization needs to embrace these generational differences to support millennial employees in achieving creativity and productivity. Still, the organization needs to inspire prospective applicants to a company (Ferri-Reed, 2014).

For a company, talent is an asset that must be maintained and developed. A consumer goods company, which is the locus of this study, has a strategy to attract, develop and retain potential talent. One of the strategies implemented by the company to attract the intention to apply for the company and retain talent is to create a new office with the concept of agility and collaboration.

As a consumer goods company, it is important to have employer branding related to company relevance. With this strategy, a brand can be successful if it is relevant to the current generation. In order to increase employer branding, one of the strategies carried out by the company is to conduct campus roadshows as an experimental tool to promote and find the young generation's interest in working in the organization.

According to a survey by SWA magazine in 2009 in Indonesia, students from leading universities remain the target of several companies, as it is common knowledge that well-known and reputable universities certainly have potential talents that are very useful for a company to accomplish its vision and mission. The company targets graduates of well-known public universities in Indonesia, such as the Universitas Indonesia and Gadjah Mada University. The Universitas Indonesia and Gadjah Mada University graduates have a high intention to work at the company being studied in this paper. This intention can be seen from the number of Universitas Indonesia and Gadjah Mada University alumni who currently work at the company. Based on LinkedIndatabase in 2019, 323 employees of the company are alumni of the Universitas Indonesia, and 161 employees are alumni of Gadjah Mada University. The alumni of Universitas Indonesia and Gadjah Mada University at the company come from various study programs. Most alumni come from the faculty of economics and business study programs and the faculty of engineering. Based on Quacquarelly Symonds (QS) (2020) data on top 15 universities in Southeast Asia, Universitas Indonesia was in the ninth position, and Gadjah Mada University was in the twelfth position. These universities outperformed one other university from Indonesia, which was included in the top fifteen of Southeast Asia's best universities. Universitas Indonesia is the best university in Indonesia; it is followed by Gadjah Mada University, the second-best university in Indonesia. Both of these universities are targets of employer branding by the company. Employer branding is essential information that companies often use to attract the best talent candidates with their efforts so that the company is the best choice for talented candidates as their place of work.

Most recent research has explored the impact of employer brand attractiveness on intention to apply (Chhabra and Sharma, 2014; Ergun and Tatar, 2016; Gomes and Neves, 2011; Ha and Luan, 2018; Saini et al., 2015; Santiago, 2019; Sharma and Prasad, 2018). However, many studies consider organizational reputation as independent variable not mediating variable (Erlinda and Safitri, 2020; Liu, 2018; Sivertzen et al., 2013). Few study consider organizational reputation as mediator (Ehsan and Nurfitri, 2021). This study tried to fill this void, because, nowadays, organizational reputation is important, especially because of the development of social media (Cable and Turban, 2003; Collins and Stevens, 2002; Erlinda and Safitri, 2020; Liu, 2018; Xie et al., 2015). This study was among the first empirical attempts to examine the role of organizational reputation in mediating the effect of employer brand attractiveness on intention to apply through data obtained from Indonesia's fresh graduate students.

Therefore, researchers discussed the issue of employer branding in increasing company attractiveness and organizational reputation, by asking questions related to employer brand attractiveness and influence on organizational reputation and the desire to apply for a job at the company among students, especially active undergraduate students, the final semester of the Faculty of Economics and Business Universitas Indonesia, Faculty of Economics and Business Gadjah Mada University and Faculty of Engineering Gadjah Mada University.

## 2 Literature review

### 2.1 Employer branding

According to Mosley and Schmidt (2017), a more recent definition of employer branding is the process of creating a distinctively great place to work and then promoting it to the talent whose knowledge and skills would help to meet its business goals and objectives. According to Ambler and Barrow (1996), an employer brand is a benefit offered by a company to employees, to create a unique identity in employees' and applicants' eyes, which can encourage them to stay together with or join a company. Branding for human resource management is called employer branding, where the brand of the employer company is seen as a “good place to work” both for the talent in the company and talent candidates. Employer branding is a term used to describe how a company communicates an offer to potential employees and employees who have worked to attract and retain their loyalty and promote the company as a different company from other companies and companies they want to work for (Backhaus and Tikoo, 2004). Employer brand is a unique and identifiable brand identity, while employer branding is the process by which a company's brand is formed and communicated both internally and externally (Sharma and Prasad, 2018). The stronger the attractiveness of employer branding, the stronger the perceived value of employer branding in applicants' perceptions. Jiang and Iles (2011) view employer branding as a “strength” that attracts the attention of applicants to the organization and encourages employees who are currently in the company to remain loyal to the company.

It is clear enough that the concept of employer branding does not change. However, the importance of it becomes crucial due to the fierce competition and the scarcity of talent. Employer branding is closely related to employee value proposition (EVP). Green (2019) defines EVP as the holistic sum of everything people experience and receive while part of a company. A strong EVP that is perceived externally attracts great people like flowers attracts bees. Thus, the most effective EVPs are those that operate as brands. That is in line with the statement of Dinnen and Alder (2017), who posit that brand perception is a key factor influencing a job seeker's impressions of what a company would be like to work for. Rampl and Kenning (2014) stated that an organization uses employer branding to attract new employees and as a means to ensure that the talent in the company is involved in the company's culture and strategy.

### 2.2 Employer attractiveness

Impressions on prospective employers, including the perception of organizational attractiveness, are the keys to success in attracting applicants (Carless, 2007). Employer attractiveness is a set of benefits expected by potential employees to work in an organization (Berthon et al., 2005). According to Altmann and Suess (2015), employer attractiveness is divided into two distinct but interrelated dimensions, namely general attractiveness, which refers to an individual's affective thinking and attitude about the company as a potential employer, and the intention to pursue work with the company actively. Employer attractiveness refers to inferences about the organization's characteristics and the related benefits perceived by potential employees that they will get by working in the organization (Reis et al., 2017).

The more positive the beliefs that job seekers have about an organization, the more likely they will be attracted to the company, and the more prepared applicants will be in applying for job vacancies (Cable and Turban, 2001). The importance of employer attractiveness for recruitment has been explored in research in HR and marketing areas. Ambler and Barrow (1996, in Gomes and Neves, 2011) have conducted research related to employer brand that shows the importance of recruiting results from a company's image as an employer. Gomes and Neves (2011) explained that employer attractiveness influences career intentions and job choice. Employer attractiveness depends on the beliefs held by job applicants in the image and the familiarity of job applicants with the organization's brand and reputation. This study measured employer attractiveness using the “EmpAt” measurement developed by Berthon (2005) that had been proven valid and reliable.

### 2.3 Organizational reputation

Reputation can be defined as a valuable strategic asset for every business. Reputation gauges the degree of trust that the consumers, clients, marketplace and the industry, as a whole, hold for a brand (Mantri, 2019 in Langham, 2019). By definition, it is clear that building a reputation is not easy. It takes years to build, but it can be destroyed in one night of the company doing something unlawful and unethical. Therefore, trust is the foundation of reputation.

Organizational reputation reflects the organization's relative position internally with talent in the company and externally with other stakeholders (Fombrun et al., 2000). According to personnel psychology, organizational reputation positively influences intention to apply for a job (Cable and Graham, 2000; Edwards, 2010). Dukerich et al. (2002) used social identity theory and stated that an organization with an attractive external image (reputation) is associated with a higher organizational identification level. In Xie et al. (2015), an organization with a more excellent perception of prestige (Mael and Ashforth, 1992) and a higher positive status (van Dick et al., 2007) will attract more identification from talent*.*

Opinium research interviewed 112 senior corporate communication in 2018, and it reveals that the benefits of an enhanced reputation are recruiting and retaining the best staff (the highest 88%). The second is more media coverage (82%), followed by a greater likelihood of receiving the benefit of the doubt from stakeholders if reputational damage is incurred (76%) (Langham, 2019). Other benefits are less significant to mention. Thomas (2019) says that reputation management is a moral duty concerning millennials, and integrity underpins their careers.

Xie et al. (2015) measured two dimensions of company reputation: the applicant's self-perception and others' perception of company reputation. Four items were adopted from a study conducted by Bergami and Bagozzi (2000) to measure organizational reputation. The four items refer to others' beliefs and beliefs of close relatives about the company that is well-known, respected, prestigious and admired*.* Even though the measurement used by Bergami and Bagozzi (2000) mainly focused on social identity in the organization, organizational identity is the personal awareness of organization members. This study assumed that organizational identity could also be applied to reflect stakeholders' perceptions (including applicant candidates). Therefore, this measurement can be adopted to measure organization reputation because it reflects how applicants perceive and think about it (Dukerich et al., 2002).

### 2.4 Intention to apply

The intention to apply for vacancies is a strong predictor in the early stages of recruitment attractiveness (Barber and Roehling, 1993) and is significant in understanding job choice applicants (Gomes and Neves, 2011). Experts in social and organizational psychologists have shown that the intention to apply for a job predicts an action (Gomes and Neves, 2011), as expressed in the theory of planned behaviour by Ajzen (1991). Therefore, if it is assumed in terms of recruitment, the intention to apply for a job can strongly predict applicants' actual decisions to apply for a job in a vacancy (Gomes and Neves, 2011). Highhouse et al. (2003) stated that the intention item is the applicant's thought about a company that explicitly implies further action to apply to a company. Therefore, the intention to move passive thoughts on a company's attractiveness is to take the job. The measurement of intention to apply was adapted from Highhouse et al. (2003) (see Table 1).

**Table 1:** Variables, definition and indicators.

Variable	Definition	Indicators
Employer Brand Attractiveness	a set of benefits expected by potential employees to work in an organization (Berthon et al., 2005)	25 items of indicator
Organizational Reputation	the organization's relative position internally with talent in the company and externally with other stakeholders (Fombrun et al., 2000)	8 items of indicator
Intention to Apply	the applicant's thought about a company that explicitly implies further action to apply to a company (Highhouse et al., 2003)	4 items of indicator

## 3 Research hypothesis

### 3.1 Employer brand attractiveness on intention to apply

According to Berthon et al. (2005), employer brand attractiveness is a benefit that future talents imagine and recognize when working for a particular organization or company. It is important as a company's strategy to attract employees with superior skills and knowledge, the primary source of competitive advantage.

Chhabra and Sharma (2014) showed a positive and significant relationship between employer branding and student intentions to apply for a job. Other research that Santiago conducted (2019) on 281 respondents (nearly 60% of respondents were millennials) showed that almost all dimensions of employer brand attractiveness influenced intention to apply for the job. Based on the results of previous studies and supporting theories, the hypothesis formulation is as follows:

H1 Employer brand attractiveness influences the intention to apply by final-year undergraduate students.

### 3.2 Organizational reputation on intention to apply

Several studies showed that one of the main determinants in recruiting talent is the organization's reputation, referring to the organization's status to other organizations (Belt and Paolillo, 1982; Cable and Turban, 2003). This study showed that an organization with a positive reputation is more attractive to job seekers. Thus, an organization's reputation acts as a brand which is an added value to work outside of the attributes of the work itself (for example, job content and salary). A study by Collins and Stevens (2002) on engineering students and their intentions to apply for an organization found that organizations' positive perceptions influence student intentions to apply for jobs in an organization. Based on the results of previous studies and supporting theories, the hypothesis formulation is as follows:

H2 Organizational reputation influences the Intention to Apply by final-year undergraduate students

### 3.3 Employer brand attractiveness on organizational reputation

Cable and Turban (2001) showed that employer brands and organizational reputation are important aspects for job seekers because job seekers see the organization's reputation as representing job and organizational attributes. This result is also supported by Sharma and Prasad (2018), which showed that an organizational reputation is one indicator that represents employer brand. Based on the results of previous studies and supporting theories, the hypothesis formulation is as follows:

H3 Employer brand attractiveness influences organizational reputation by final-year undergraduate students.

### 3.4 Employer brand attractiveness on intention to apply through organizational reputation as a mediating variable

**Figure 1:**
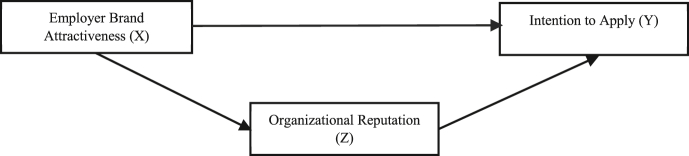
Research model.

According to Sivertzen et al. (2013), employer branding is an important factor in increasing employer attractiveness and intention to apply for a job. In that study, employer brand attractiveness is divided into five dimensions: application value, development value, economic value, interest value and social value (Sivertzen et al., 2013). Sivertzen et al. (2013) continued a study on employer branding and included organizational reputation, social media use and intention to apply variables in a model with employer attractiveness dimensions. According to Collins and Han (2004), studies showed the relationship between an organizational reputation and how it attracts applicants. Furthermore, Ehsan and Nurfitri (2021) found that organization reputation significant mediate the relationship between employee brand attractiveness and intention to apply. Based on previous studies and supporting theories, the hypothesis formulation is as follows (see Figure 1):

H4 Employer Brand Attractiveness influences the Intention to Apply by final-year undergraduate students through organizational reputation as a mediating variable*.*

## 4 Research method

This study applied a quantitative approach. According to Sekaran (2016), quantitative research is research by obtaining data in numbers or qualitative data that is transformed into numbers. Based on the research objectives, this study used explanatory research. These data were taken only at one time, namely in November 2019, so this study was a cross-sectional study. This study's population was the Universitas Indonesia undergraduate students of the Faculty of Economics and Business and the Faculty of Engineering and Gadjah Mada University undergraduate students of the Faculty of Economics and Business and the Faculty of Engineering. The sample in this study was the Universitas Indonesia batch 2016 bachelor students comprised of the Faculty of Economics and Business and at least batch 2016 or 7th-semester bachelor students of Gadjah Mada University Engineering and the Faculty of Economics and Business.

The sampling technique used accidental sampling or convenience sampling. Convenience sampling is a non-probability sampling technique where samples are selected from the population simply because they are available to researchers. Researchers carried out two techniques when conducting this research, namely based on primary data and secondary data. Primary data are data obtained directly and through surveys and in-depth interviews, while secondary data are obtained through data other parties have published. Primary data collection used a survey. Questionnaires were distributed offline and online to 425 respondents through the Google Form application. Respondents consisted of students of the Universitas Indonesia and Gadjah Mada University. In the study of employer brand attractiveness on the intention to apply through organizational reputation as a mediating variable and the primary data from the survey needed, researchers also need supplementary data and information in the form of secondary data. Secondary data needed by researchers were from journals, books, sites and literature studies.

Employer brand attractiveness was measured in five dimensions by Berthon (2005): interest value, social value, economic value, development value and application value. Organizational reputation was measured by using theory from Bergami and Bagozzi (2000), which consisted of eight question items. Finally, the intention to apply was measured by using theory from Highhouse et al. (2003), consisting of four question items. The applicants were asked whether te had the intention to apply to one of the well-known consumer goods companies in Indonesia. These items were measured by using the interval-Likert scale of 1–5, where 1 represents “Strongly Disagree”, and 5 represents “Strongly Agree”. Respondents were asked to choose one option that best suited to their condition. It is often used to measure respondents’ perceptions whether they agree or disagree with particular statements.

This study's hypothesis testing was conducted using simple regression analysis to test hypothesis 1, hypothesis 2 and hypothesis 3 and multiple regression analysis to test hypothesis 4. This study used multiple tests to test the effect of mediating variable.

This study did not have ethical approval because in the Faculty of Administrative Sciences Universitas Indonesia, there is no formal board with authority to give ethical approval for research carried out by lecturers of the Faculty of Administrative Sciences Universitas Indonesia. However, informed consent for all participants in this study was distributed along with the questionnaire.

## 5 Analysis

### 5.1 Validity and reliability test

Validity and reliability test was used to ensure the valid and reliable variable measurement to be used and analysed further. Validity test used corrected item-total correlation (r corrected) and exploratory factor analysis with Kaiser-Meyer-Olkin (KMO) test and Barlett's test of sphericity. Corrected item-total correlation (r corrected) shows that correlation value greater than 0.3 or significant at 5%. This means all indicators used in this study are valid Indicators' Validity test results can be seen in Table 2.

**Table 2:** Indicator's validity test.

Variable	Indicator	Corrected Item-Total Correlation	Decision
Employer Brand Attractiveness	IV 1	.435	Valid
	IV 2	.512	Valid
	IV 3	.525	Valid
	IV 4	.394	Valid
	IV 5	.536	Valid
	SV 1	.521	Valid
	SV 2	.549	Valid
	SV 3	.574	Valid
	SV 4	.554	Valid
	SV 5	.534	Valid
	EV 1	.536	Valid
	EV 2	.491	Valid
	EV 3	.530	Valid
	EV 4	.508	Valid
	EV 5	.529	Valid
	DV 1	.583	Valid
	DV 2	.646	Valid
	DV 3	.656	Valid
	DV 4	.594	Valid
	DV 5	.628	Valid
	AV 1	.574	Valid
	AV 2	.515	Valid
	AV 3	.450	Valid
	AV 4	.481	Valid
	AV 5	.572	Valid
Organizational Reputation	OR 1	.464	Valid
	OR 2	.492	Valid
	OR 3	.617	Valid
	OR 4	.623	Valid
	OR 5	.449	Valid
	OR 6	.519	Valid
	OR 7	.554	Valid
	OR 8	.563	Valid
Intention to Apply	IA 1	.502	Valid
	IA 2	.563	Valid
	IA 3	.478	Valid
	IA 4	.517	Valid

Meanwhile, KMO's value shows that indicators that are used to measure employer branding, organization reputation and intention to apply are greater than 0.5, while corrected item-total correlation (r corrected) shows that correlation value is greater than 0.3 or significant at 5%. This result indicates sufficient items for each factor. Barlett's test of sphericity's value of the indicators is used to measure employer branding, organization reputation and intention to apply; it shows they are significant (less than 0.05). This study used Cronbach's alpha to measure reliability. The value of Cronbach's alpha for all three variables tested is above 0.7. This value indicates the internal consistency of the items is good—the results of validity and reliability test are shown in Table 3.

**Table 3:** Variable validity and reliability test.

Variable	KMO Test Value	Barlett's Test of Sphericity Significant Value	Alpha Cronbach
Employer Branding	0.918	0.000	0.923
Organization Reputation	0.857	0.000	0.892
Intention to apply	0.827	0.000	0.869

Source: Processed data (2019).

### 5.2 Descriptive analysis

Descriptive analysis based on gender showed that 53.2% of respondents were male, and 46.8% were female. Additionally, it can be seen that respondents in this study were dominated by respondents aged 21 years with a percentage of 67.1%, followed by those aged 20 years with a percentage of 14.8%, those aged 22 years with a percentage of 13.2%, and those aged 23 years and 19 years with a percentage of 2.4% and those aged 25 years with a percentage of 0.2%. This study's sample was the Universitas Indonesia students (51.8%) and Gadjah Mada University students (48.2%). Furthermore, respondents in this study were Faculty of Engineering Gadjah Mada University students with a percentage of 33.2%, followed by Faculty of Engineering Universitas Indonesia students with a percentage of 31.8%, Faculty of Economics and Business Universitas Indonesia students with a percentage of 20% and students of the Faculty of Economics and Business Gadjah Mada University with a of 15.1%.

### 5.3 Inferential analysis

From the results of the calculations, the first criterion of the mediation variable accepted hypothesis null. Based on simple linear regression analysis, employer brand attractiveness (X) as an independent variable had a significant influence on the intention to apply (Y) as the dependent variable and employer brand attractiveness (X) as an independent variable had a significant influence on the organizational reputation (Z) as a mediating variable with simple regression analysis. The third criterion cannot be fulfilled based on the results of multiple linear regression, which showed that organizational reputation (Z) as a mediating variable had no significant influence on the intention to apply (Y) as a dependent variable in controlling employer brand attractiveness (X) as an independent variable which influenced intention to apply. The fourth criterion can be fulfilled, where the influence of employer brand attractiveness (X) on the intention to apply (Y), which had a significant influence in the results of simple linear regression analysis, remains significant after being controlled by organizational reputation (Z) variable in multiple linear regression analysis with smaller influence value (see Table 4).

**Table 4:** Results of simple and multiple linear regression.

Independent Variable	Dependent Variable	Regression Test	B	t-calculation	Sig.	Interpretation
*Employer Brand Attractiveness* (X)	*Intention to Apply (Y)*	Simple Linear	0.132	12.113	0.000	significantly influence (Ho Rejected)
*Employer Brand Attractiveness* (X)	*Organizational Reputation* (Z)	Simple Linear	0.207	14.876	0.000	significantly influence (Ho Rejected)
*Organizational Reputation* (Z)	*Intention to Apply* (Y)	Multiple Linear	0.057	1.511	0.131	not significantly influence (Ho Accepted)
*Employer Brand Attractiveness* (X)	*Intention to Apply* (Y)	Multiple Linear	0.120	8.944	0.000	significantly influence (Ho Rejected)

Source: Processed data (2019).

These results indicate that if first to third criteria can be met, then organizational reputation can mediate the influence of employer brand attractiveness of company on the intention to apply to a company partially. However, because the third criterion was not fulfilled, organizational reputation cannot be a mediating variable between the influence of employer brand attractiveness on the intention to apply.

Researchers used the Sobel test to estimate the indirect effect of the independent variable (X) on the dependent variable (Y) through a mediating variable (Z). Researchers conducted a significance test of the fourth hypothesis or organizational reputation as a mediating variable. Researchers with the following results used the Sobel test: (see Table 5)

**Table 5:** Results of *sobel test*.

Sobel Test Statistic	Std. Error	p-Value
1,49234011	0,00790637	0.13561001

Source: Processed data (2019).

Based on the fourth hypothesis significance test results above, there was no significant influence of a mediating variable. The result is indicated by the Sobel test statistic (t_-value_) of 1.492, which did not meet the criteria because it was smaller than (t_-table_) of 1.96 and the significance value (P-value) of 0.136, which did not meet the criteria because it was greater than 0.05. In conclusion, organizational reputation did not mediate the relationship between employer brand attractiveness and the intention to apply*.*

## 6 Discussion

Based on the results in this study through regression test, employer brand attractiveness has a moderate influence on intention to apply. Furthermore, employer brand attractiveness also has a moderate influence on organizational reputation. Meanwhile, organizational reputation has a weak influence on the intention to apply. However, based on significant value, organizational reputation has stronger impact on intention to apply as compared to employer brand attractiveness. In this study, the significance of organizational reputation as a mediator between employer brand attractiveness and intention to apply is not found.

Respondents to our research were mainly gen millennials. Every generation has its lifestyle and work values. Millennials' main characteristics are achievement-focused, work-life balance, career development and advancement, work that has meaning and social values through corporate social responsibility (De Hauw and De Vos, 2010; Ng and Gosset, 2013; Smith and Nichols, 2015). On the other side, technology shapes millennials' values and characteristics. According to Gallup Global Study (Clifton, 2019), millennials are highly networked and prefer to go straight to the source of companies they are interested in. They also cast a wide net in their job search from many online sources such as professional network sites, employee ranking sites and general search engines to explore many options. The result of data analysis and processing shows that role of mediating variable had no significance on the Sobel test value (t_-value_) of 1,492, which did not meet the criteria because it was smaller than (t_-table_) of 1.96. Based on these results, organizational reputation did not mediate the influence of employer brand attractiveness on the intention to apply. It proves that the company has a strong employer brand attractiveness so that it can form an intention to apply without considering the organizational reputation. The company has successfully promoted its employer branding through many kinds of programs that fit the millennials' values, such as the future leader's program, promoting world development advancement, creating a social impact of business and quality of self-development. Those programs have a significant and direct impact on the intention to apply.

As stated by Witzel (2018), our reputation and our brand is not determined by ourselves, or at least not solely by ourselves. Stories tell whether companies have a good or bad reputation. If people tell good stories, company's reputation is positive. On the other hand, bad reputation happens when people start to tell bad things about company such as treating employees unfairly, unethical behaviour toward environment, cheating customers or paying employees below minimum regulation standard. Actions speak louder than words; this means that company should communicate its employment branding through varies social media to attract prospective talents, and this branding must be consistent with its promise. In other words, employer brand promise should be matching with reality. When the company spreads its positive employment branding through media intensively, it becomes more attractive to employment seekers.

In a radical transparency nowadays, social media is a means for anyone to know about a company's action. Therefore, with intensive corporate communication, employer branding can directly influence on intention to apply. Employer branding surely has a positive impact on reputation; however, with the intensive corporate communication throughout media, it becomes more important to employment seeker to get attracted through organizational reputation.

Although, in this study, organizational reputation did not mediate the influence of employer brand attractiveness on the intention to apply because the organization's reputation was included in the company's employer branding through interest values that reflect the company reputation. Through this discussion, the company's management can make the results of this study as a reference to pay attention to factors that can increase intention to apply, and the company can maintain things that are already good in the company, such as employer branding to maintain the reputation of the company.

The findings in this study support previous research Chhabra and Sharma (2014); Ergun and Tatar (2016); Gomes and Neves, 2011; Ha and Luan (2018); (Saini et al., 2015)Saini et al., 2015; Santiago (2019); Sharma and Prasad (2018); Larsson and Rosell (2014), which explain that employer brand attractiveness has a positive effect on intention to apply. The result is also in line with Collins and Stevens (2002), Erlinda and Safitri (2020), Liu (2018), Cable and Turban (2003)(Cable and Turban, 2003) and Xie et al. (2015)(Xie et al., 2015); they explain that organizational reputation has significantly affects intention to apply. However, whenever organizational reputation is considered as a mediator, the result does not fall in line with previous research (Ehsan and Nurfitri, 2021) because in the eyes of millennial generation, they consider employer brand attractiveness more than organizational reputation, as explained in the previous paragraph.

## 7 Conclusion

This study aimed to answer all the problem formulations that have been proposed previously. The conclusions of this study are as follows: first, employer brand attractiveness significantly influences intention to apply with a positive relationship; second, employer brand attractiveness had a significant influence on organizational reputation with a positive relationship and last, organizational reputation had a significant influence on intention to apply with a positive relationship, and organizational reputation as a mediator had no significant influence between employer brand attractiveness and intention to apply. It can be concluded that organizational reputation does not mediate the impact of employer brand attractiveness on intention to apply. Indonesia's fresh graduate students do not consider organizational reputation when they search for a company to apply. They prefer the values that the company offers. The company should pay more attention to applicants' value to attract potential talent. Furthermore, the values offered by the company are essential to build a robust organizational reputation.

Based on the conclusion above, the following are managerial implications that can be taken as a strategy in attracting millennials’ intentions to apply. A study on 425 respondents consisting of final-year students of the Universitas Indonesia and Gadjah Mada University showed that employer brand attractiveness in the company could lead to the intention to apply without considering organizational reputation variables. However, the company already had a good employer brand attractiveness so that it had a positive impact on the company, one of which is the improvement of organizational reputation and intention to apply. Based on the results of this study, there are several recommendations to improve organizational reputation and intention to apply; for example, in employer brand attractiveness, there are five dimensions; interest value is rated the highest with respect to the degree to which a person is attracted to an employer organization that can provide a challenging work environment, appreciate the creativity of employees and recognize that the company has high-quality products. Therefore, the company needs to focus on interest value to increase its reputation and the intention to apply to the company. The company needs to maintain and even increase socialization activities to universities, especially reputable universities and prestigious universities, to obtain quality talents that meet company criteria.

## 8 Limitations and suggestions for further study

This study has some limitations and opportunity for further study that can be fulfilled. Data collection was conducted in Indonesia and had a limited number of samples, so the results may not be generalized to other potential employees outside Indonesia. However, the results of our model provided some preliminary support for the relationships between employer brand attractiveness, organization reputation and intention to apply. Further studies are expected to increase the number of samples, especially at universities with a good reputation and high ranking, to get talent access to these renowned universities.

## Declarations

### Author contribution statement

Pantius D. Soeling: Conceived and designed the experiments; Analyzed and interpreted the data; Wrote the paper.

Sesilia Dhea Ajeng Arsanti: Contributed analysis tools or data.

Fibria Indriati: Analyzed and interpreted the data; Wrote the paper.

### Funding statement

This work was supported by the Faculty of Administrative Science Universitas Indonesia through Research Grant (namely Hibah Jabfung)

### Data availability statement

Data will be made available on request.

### Declaration of interests statement

The authors declare no conflict of interest.

### Additional information

No additional information is available for this paper.
